# Four-year maintenance treatment with adalimumab in Japanese patients with moderately to severely active ulcerative colitis

**DOI:** 10.1007/s00535-017-1325-2

**Published:** 2017-03-20

**Authors:** Yasuo Suzuki, Satoshi Motoya, Hiroyuki Hanai, Toshifumi Hibi, Shiro Nakamura, Andreas Lazar, Anne Martin Robinson, Martha Skup, Nael Mohamed Mostafa, Bidan Huang, Roopal Thakkar, Mamoru Watanabe

**Affiliations:** 1 Toho University Medical Center Sakura Hospital, Chiba, Japan; 2 IBD Center, Sapporo Kosei General Hospital, Sapporo, Japan; 3 Hamamatsu South Hospital, Shizuoka, Japan; 4 IBD Centre, Kitasato University, Kitasato Institute Hospital, Tokyo, Japan; 5 Hyogo College of Medicine Hospital, Nishinomiya, Hyogo Japan; 6 AbbVie Deutschland GmbH & Co. KG, Ludwigshafen, Germany; 7 AbbVie Inc., North Chicago, IL USA; 8 Department of Gastroenterology, Tokyo Medical and Dental University, Tokyo, Japan

**Keywords:** Clinical remission, Tumor necrosis factor

## Abstract

**Background:**

The 52-week safety and efficacy of adalimumab in Japanese patients with moderately to severely active ulcerative colitis were demonstrated in a placebo-controlled phase 2/3 trial. Data from patients who enrolled in the open-label extension study are presented.

**Methods:**

Remission and response per the full Mayo score (FMS) and the partial Mayo score (PMS), remission per the Inflammatory Bowel Disease Questionnaire (IBDQ) score, corticosteroid-free remission, and mucosal healing were assessed up to week 196 (week 208 for remission/response per PMS) of adalimumab treatment in patients who received one or more doses of adalimumab with use of a hybrid nonresponder imputation (hNRI) method. Nonresponder imputation was used for missing data up to the latest possible follow-up date for each patient, followed by observed case. Adalimumab trough concentrations were reported from week 52 to week 196 of treatment. Treatment-emergent adverse events were reported for all adalimumab-treated patients.

**Results:**

Two hundred sixty-six patients received adalimumab. At week 196 of treatment, remission and response rates per FMS, remission and response rates per PMS, remission rate per IBDQ score, mucosal healing rate, and corticosteroid-free remission rate were 19.2%, 32.2%, 22.5%, 32.5%, 33.1%, 30.5% (hNRI), and 40.5% (17/42; as observed), respectively. Serum adalimumab concentrations remained constant in patients receiving 40 mg every other week but increased in patients who underwent dose escalation. The safety profile was consistent with that in the 52-week study.

**Conclusions:**

The efficacy of adalimumab in Japanese patients with moderately to severely active ulcerative colitis was maintained for up to 4 years of treatment. No new safety signals were observed.

**Electronic supplementary material:**

The online version of this article (doi:10.1007/s00535-017-1325-2) contains supplementary material, which is available to authorized users.

## Introduction

Ulcerative colitis is an inflammatory bowel disease in which inflammation of the colonic mucosal surface is characterized by bloody diarrhea, urgency, tenesmus, abdominal pain, and fever in some cases [1]. Patients experience periods of relapse and remission, although many patients have chronically active disease, with substantial impact on quality of life [1–4].

The incidence and prevalence of inflammatory bowel diseases such as ulcerative colitis and Crohn’s disease are lower in Asian countries than in the West but are rapidly increasing, especially in East Asia and Japan [5–7]. The prefecture prevalence of ulcerative colitis and Crohn’s disease in Japan in 2011, when this study began, ranged from 64.8 to 117.4 per 100 000 person-years [8]. Global estimates of ulcerative colitis prevalence range from 4.9 to 505 in Europe, from 37.5 to 248.6 in North America, and from 4.9 to 168.3 in Asia and the Middle East [9].

Elevated levels of tumor necrosis factor α (TNFα) in the serum, stools, and mucosa of patients with ulcerative colitis suggest a prominent role for this inflammatory cytokine in the pathogenesis of ulcerative colitis, a premise supported by the effectiveness of anti-TNFα treatments [1, 10–12]. The 52-week efficacy and safety of adalimumab, a fully human monoclonal antibody that targets TNFα, in Western patients with moderately to severely active, treatment-refractory ulcerative colitis were demonstrated in the phase 3 studies ULTRA 1 and ULTRA 2 [13–16]. A long-term open-label extension study that enrolled patients from both of these studies (ULTRA 3) demonstrated that the efficacy and safety of adalimumab are maintained for up to 208 weeks of treatment [17].

The safety and efficacy of adalimumab in Japanese patients with moderately to severely active ulcerative colitis were demonstrated in a 52-week phase 2/3 study [18]. In this report, we present the results of up to 208 weeks of adalimumab treatment in all patients who received one or more doses of adalimumab in the phase 2/3 study and an open-label extension study, studies conducted to evaluate the long-term efficacy and safety of adalimumab in Japanese patients with moderately to severely active ulcerative colitis.

## Methods

### Study design

Detailed information about the design of the 52-week phase 2/3 study and patient disposition in that study have been published [18]. Briefly, the 52-week phase 2/3 study was a randomized, double-blind, placebo-controlled trial evaluating the efficacy, safety, and pharmacokinetics of adalimumab as induction (weeks 0–8) and maintenance (from week 8) therapy in Japanese patients aged 15 years or older who had biopsy-confirmed, moderately to severely active ulcerative colitis (i.e., Mayo score 6–12; endoscopy subscore 2 or greater) despite concurrent treatment with oral corticosteroids or immunomodulators (or both) [18]. Patients (*N* = 274) were randomized 1:1:1 to receive 160 mg adalimumab at week 0, 80 mg adalimumab at week 2, and 40 mg adalimumab every other week from week 4 (160/80 arm); 80 mg adalimumab at week 0, 40 mg adalimumab at week 2, and 40 mg adalimumab every other week from week 4 (80/40 arm); or placebo. Patients in any study arm who had an inadequate response at or after week 8 could move to an open-label rescue arm [18]. Rescue treatment consisted of 4 weeks of blinded adalimumab therapy (160 mg initially and 80 mg 2 weeks later for patients in the placebo arm, or 40 mg initially and 2 weeks later for patients in either adalimumab arm) followed by open-label adalimumab 40 mg therapy every other week, with the possibility of escalation to 80 mg every other week in the case of inadequate response or disease flare.

Patients who received placebo or adalimumab (in either the double-blind phase or the rescue arm) and completed the 52-week study received open-label adalimumab 40 mg therapy every other week from week 52 until approval of adalimumab therapy for ulcerative colitis by Japanese authorities during an open-label extension study. On entry into the extension study, patients whose treatment was escalated to 80 mg adalimumab every other week by week 52 continued with the same dose. Self-injection of the study drug was allowed after week 52, as appropriate. During the open-label extension study, escalation of the adalimumab dose to 80 mg every other week was allowed at or after week 60 from the lead-in study baseline for patients experiencing inadequate response or disease flare. Inadequate response was defined as a partial Mayo score (PMS; i.e., the Mayo score without the endoscopy subscore) greater than or equal to the baseline PMS on two consecutive visits at least 14 days apart for patients with a baseline PMS of 3–7, or PMS of 7 or greater on two consecutive visits at least 14 days apart for patients with a baseline PMS of 8 or 9. Disease flare was defined as a PMS difference of 3 or greater compared with PMS at week 52 or at the last evaluation before the disease flare on two consecutive visits at least 14 days apart. Patients who continued to have inadequate response or disease flare while receiving 80 mg adalimumab every other week were to be withdrawn from the study.

### Efficacy assessments

The baseline for this analysis was defined as the day of the first adalimumab dose for each patient. Long-term efficacy was analyzed from the first dose of adalimumab up to 196 weeks of treatment in all patients who received one or more doses of adalimumab in the phase 2/3 study or the open-label extension study (the “any ADA” set). This analysis set included patients who were randomized to receive placebo but who received adalimumab as rescue therapy during the phase 2/3 lead-in study or during the open-label extension study. Efficacy end points were remission defined by the full Mayo score (FMS; 2 points or less with no individual subscore greater than 1 point), remission defined by PMS (2 points or less with no individual subscore greater than 1 point), and remission defined by the Inflammatory Bowel Disease Questionnaire (IBDQ; score 170 points or greater); change from the baseline in the IBDQ, response by FMS [decrease in FMS of 3 points or more and 30% or more from the baseline plus a decrease in the rectal bleeding subscore (RBS) of 1 point or more or an absolute RBS of 0 or 1], and response by PMS (decrease in PMS of 2 points or more and 30% or more from the baseline plus a decrease in RBS of 1 point or more or an absolute RBS of 0 or 1); and mucosal healing (endoscopy subscore 1 or less). The change from the baseline in the mean physical and mental summary scores of the 36-item short-form quality-of-life assessment (SF-36) was also reported for the any ADA set. Discontinuation of corticosteroid therapy and corticosteroid-free remission (Mayo score 2 or less with no individual subscore greater than 1 and discontinuation of corticosteroid therapy) were assessed in patients who received corticosteroids at the baseline of the lead-in phase 2/3 study (*n* = 175). An additional analysis was performed on the any ADA set for remission and response by FMS, remission and response by PMS, and mucosal healing, whereby patients were imputed as nonresponders at the time of escalation to dosing at 80 mg every other week. These data are reported as “no dose escalation.”

During the double-blind study, PMS was evaluated at weeks 2, 4, 6, and 8, and monthly thereafter, and FMS was evaluated at weeks 8, 32, and 52 [18]. During the open-label extension study (i.e., after week 52), PMS was evaluated every 12 weeks and at the completion or early termination visit. Because endoscopy was performed every 48 weeks during the open-label extension study, FMS was also evaluated every 48 weeks and at the completion or early termination visit. Stool frequency subscore and RBS were evaluated on the basis of patients’ diary entries, with use of the worst diary entry from the 3 days before each study visit for each patient-reported subscore. IBDQ and SF-36 scores were evaluated every 24 weeks after week 52 and at the completion or early termination visit.

### Pharmacokinetics and anti-adalimumab antibodies

Methods for the assessment of pharmacokinetics and anti-adalimumab antibodies (AAAs) have been described [18]. In the open-label extension study, blood samples were obtained for evaluation of serum adalimumab and AAAs every 24 weeks after week 52, at the completion or early termination visit, and at the 28-day follow-up visit. Samples were assessed by use of a validated enzyme-linked immunosorbent assay based on a double-antigen technique. The lower limit of quantitation for adalimumab was 3.13 ng/mL in diluted serum and 31.3 ng/mL in undiluted serum. AAA was measured in serum samples in which the adalimumab concentration was less than 20 ng/mL. The lower limit of quantitation for AAA was 1.0 ng/mL in diluted serum and 10 ng/mL in undiluted serum. Trough serum concentrations over time from week 52 of the lead-in study were summarized by dose-escalation status (i.e., continuing with 40 mg every other week versus escalation to 80 mg every other week) and by AAA status for patients randomized to receive adalimumab in the lead-in study and who entered the open-label extension study.

### Safety assessments

Adverse events and changes in laboratory variables and vital signs were monitored throughout the study. During the open-label extension study (after week 52), vital signs were recorded and physical examination and laboratory tests performed every 12 weeks; chest X-rays (as part of tuberculosis screening and pregnancy testing) were obtained every 24 weeks.

### Statistical methods

Because patients in the any ADA cohort received their first adalimumab dose at different times and therefore had various durations of follow-up, the categorical efficacy end points were analyzed by use of a hybrid nonresponder imputation (hNRI) method. This was used because use of standard nonresponder imputation would not have accurately reflected the long-term efficacy of adalimumab in patients with ulcerative colitis who discontinued participation in the study per protocol because of the approval of adalimumab in Japan and not for clinical reasons. The latest possible visit for an individual patient in the study was based on the time from his/her first dose of adalimumab to the date of the approval of adalimumab for treatment of ulcerative colitis in Japan on June 14, 2014; consequently, the follow-up time was not the same in all patients. In the hNRI analysis, a patient was considered not to have efficacy if he/she discontinued participation in the study or had missing data up to his/her latest possible visit. Afterward, an observed-case analysis was applied (i.e., patients were not included in the nonresponder imputation analysis at visit weeks beyond their latest possible visit). This resulted in a decrease of the denominator for binary end point calculations in the any ADA cohort after week 112.

Patients whose dose was escalated to 80 mg adalimumab every other week were not imputed as nonresponders for efficacy unless specified otherwise (“no dose escalation”). The change from the baseline in the physical and mental components of SF-36, the change from the baseline in the IBDQ score, discontinuation of corticosteroid therapy, and corticosteroid-free remission were reported as observed. For the pharmacokinetic analysis, adalimumab concentration is summarized by treatment group at each time point by use of descriptive statistics, including the number of patients; the number of nonmissing observations; mean, median, and standard deviation; coefficient of variation; and minimum, maximum, and geometric mean. Serum AAA concentrations are listed by treatment group at each collection time.

## Results

### Patient disposition and baseline characteristics

**Fig. 1:**
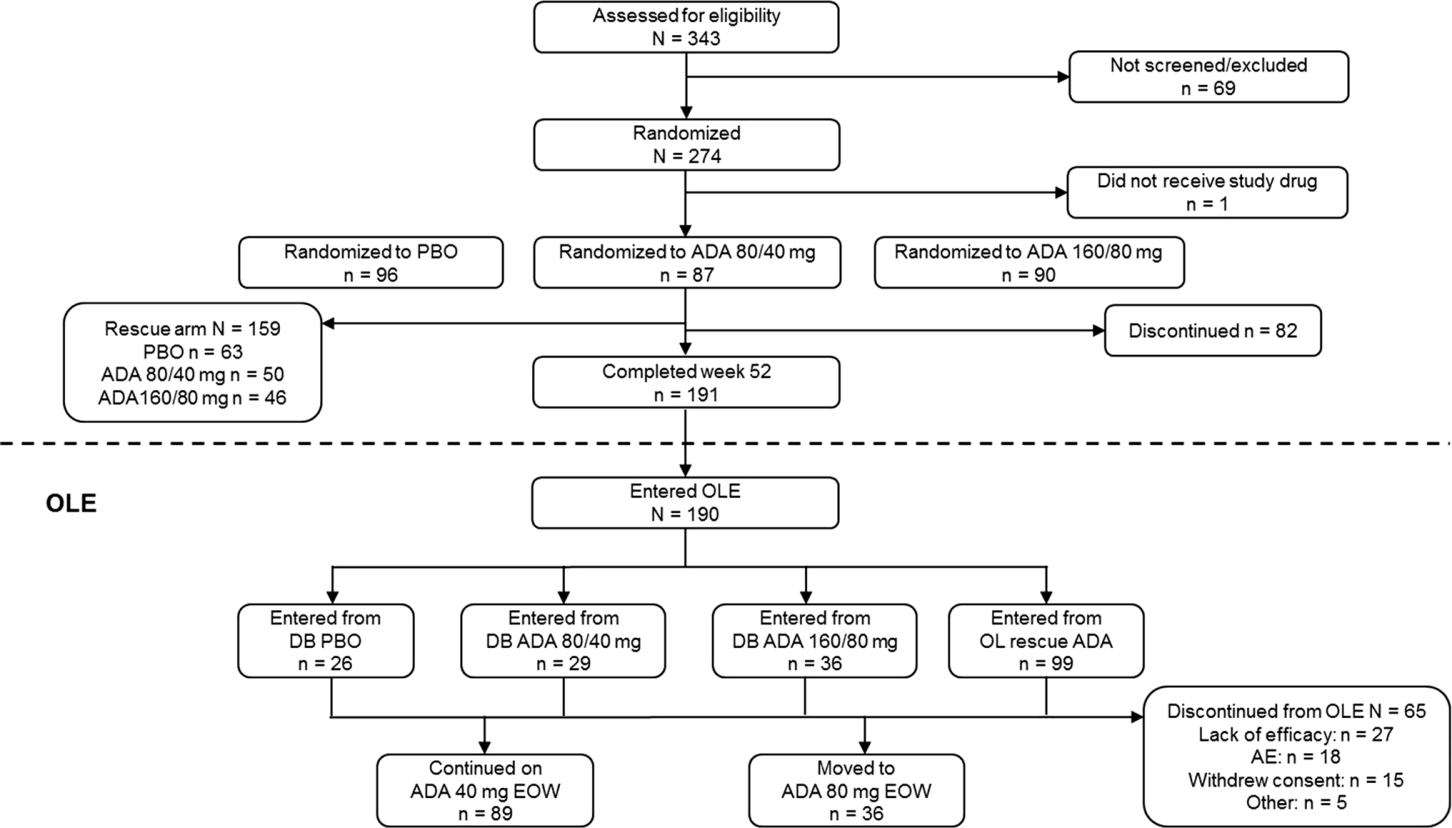
Patient disposition. *ADA* adalimumab, *AE* adverse event, *DB*, double blind, *EOW* every other week, *OL* open-label, *OLE* open-label extension, *PBO* placebo

The disposition of the patients in the double-blind and extension periods of the study is shown in Fig. 1. Two hundred sixty-six patients received adalimumab (any ADA set) either during the double-blind period (177 patients randomized to receive adalimumab and 63 randomized to receive placebo who received adalimumab in the rescue arm) or the open-label extension period, in which 26 patients received adalimumab for the first time. Of the 191 patients who completed the lead-in study to week 52, 190 entered the open-label extension study (91 patients who had completed the double-blind therapy and 99 patients who had completed the open-label adalimumab therapy); 65 patients discontinued participation during the open-label extension study, most commonly because of lack of efficacy (27 patients). A total of 42.1% of patients (112/266) in the any ADA set received adalimumab at 80 mg every other week during the lead-in study or the open-label extension study. Of the 266 patients in the any ADA set, 119 patients (44.7%) completed the study.

The demographics and patient characteristics in the any ADA set at the time of enrollment in the lead-in study are shown in Table 1. Most patients were male (175/266; 65.8%), the mean age was 42.6 years (range 15–74 years), and 64.3% of patients had pancolitis. The mean duration of ulcerative colitis was 8.1 years (range 0.4–37.8 years), and the mean FMS and PMS were 8.5 and 6.1, respectively, consistent with a diagnosis of moderate-to-severe ulcerative colitis.

**Table 1:** Demographics and clinical characteristics at lead-in study enrollment of all patients in the “any ADA” set (*N* = 266)

	Value
Age (years)^a^	42.6 (14.4)
Sex, male	175 (65.8%)
Disease duration (years)^a^	8.1 (7.2)
Site of ulcerative colitis	
Entire large intestine	171 (64.3%)
Descending colon	92 (34.6%)
Other^b^	3 (1.1%)
Mayo score^a^	8.5 (1.46)
Partial Mayo score^a^	6.1 (1.3)
Ulcerative colitis disease activity index score^a^	8.5 (1.5)
IBDQ score^a,c^	146.4 (29.6)
SF-36 score^a,d^	
Physical component	45.2 (7.2)
Mental component	39.4 (11.6)
C-reactive protein (mg/dL)^e^	0.3 (0.1–10.8)
Concomitant medication	
Aminosalicylate	249 (93.6%)
Corticosteroids	175 (65.8%)
Immunomodulators	
Azathioprine	109 (41.0%)
6-Mercaptopurine	22 (8.3%)

*IBDQ* Inflammatory Bowel Disease Questionnaire

^a^The mean is given, with the standard deviation in *parentheses*

^b^Distal colitis and appendix or ascending colon, colon or rectal colon or transverse colon, and rectal to sigmoid colon

^c^ *N* = 262

^d^ *N* = 265 (missing result for one patient)

^e^The median is given, with the range in *parentheses*

### Remission, response, and mucosal healing over time

**Fig. 2:**
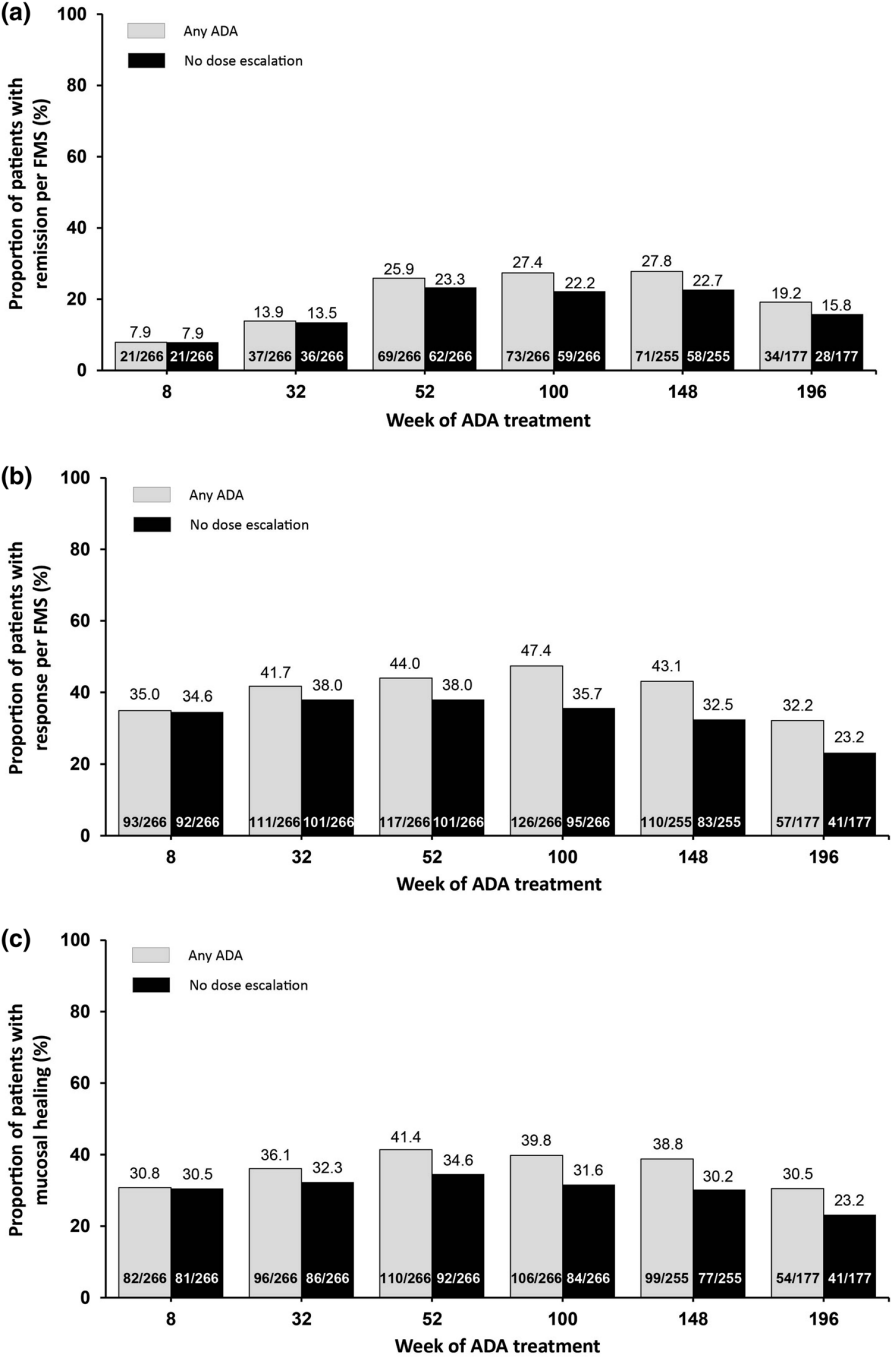
Efficacy of adalimumab over time for patients regardless of dose in the “any ADA” set and imputing patients whose treatment was escalated to 80 mg every other week as nonresponders (“no dose escalation”). Rates of **a** remission per full Mayo score (FMS), **b** response per FMS, and **c** mucosal healing (endoscopy subscore 1 or less) (hybrid nonresponder imputation; *N* = 266). *ADA* adalimumab

Remission (per FMS), response (per FMS), and mucosal healing were achieved early and were sustained throughout the study (Fig. 2). Data are presented from the first adalimumab dose to week 196 for the end points based on FMS or PMS. At week 196 of adalimumab treatment, hNRI analysis in the any ADA set gave rates of remission and response per FMS of 19.2% (34/177) and 32.2% (57/177) respectively (Fig. 2a, b); the rates of remission and response per PMS were 22.5% (27/120) and 32.5% (39/120) respectively (Fig. S1). When patients were imputed as nonresponders at the time of moving to 80 mg every other week, the rates of response at week 196 of treatment per FMS and PMS by hNRI analysis were 23.2% (41/177) and 22.5% (27/120), respectively (Figs. 2b, S1b; no dose escalation). In addition, PMS data were collected up to week 208; PMS remission and response rates were 26.5% (22/83) and 34.9% (29/83), respectively, by hNRI analysis in the any ADA set, and when patients whose treatment was escalated to 80 mg every other week were imputed as nonresponders, the PMS response rate was 24.1% (20/83) by hNRI analysis (data not shown).

The proportion of patients with mucosal healing remained stable from week 8 through week 196 of treatment (30.8–30.5%) (Fig. 2c).

### Corticosteroid-free remission

**Fig. 3:**
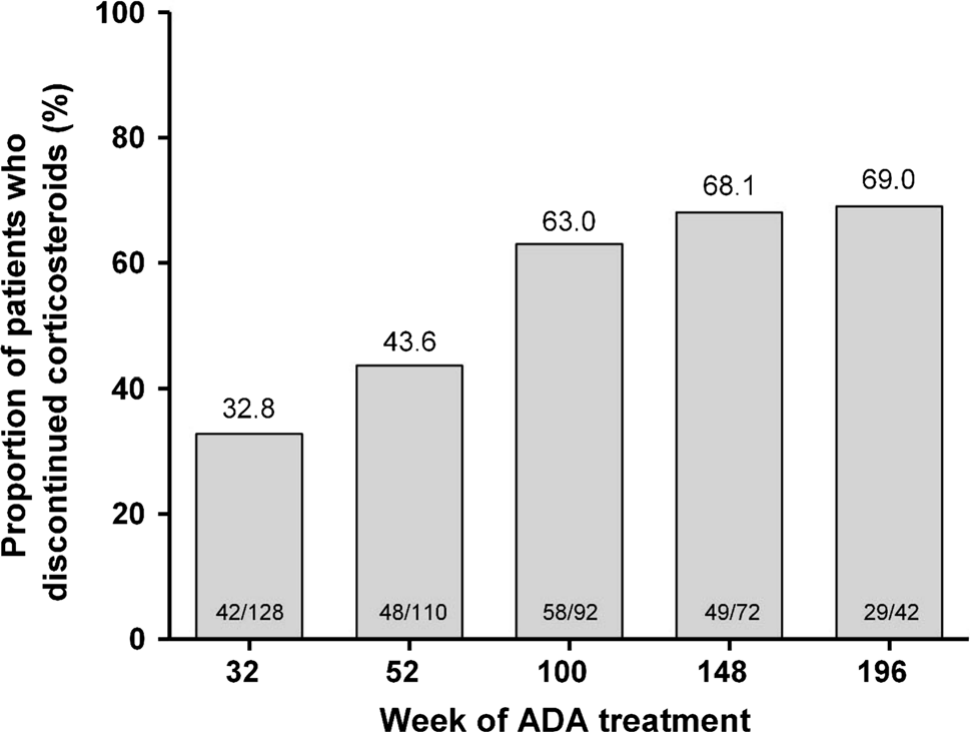
Proportion of patients who used corticosteroids at lead-in study enrollment and who discontinued corticosteroid therapy over time (observed analysis; *N* = 175). *ADA* adalimumab

In the any ADA set, 65.8% of patients (175/266) used corticosteroids at the lead-in study baseline. From week 32 through week 196 of adalimumab treatment, the proportion of patients who discontinued corticosteroid therapy increased from 32.8% (42/128) to 69.0% (29/42; observed analysis) (Fig. 3). The corticosteroid-free remission rate in patients in the any ADA set who used corticosteroids at the lead-in study baseline increased from 10.2% (13/128 patients) at week 32 to 38.0% (35/92) at week 100 and remained stable through week 196 of adalimumab treatment [40.5% (17/42); observed analysis].

### Health-related quality of life

**Fig. 4:**
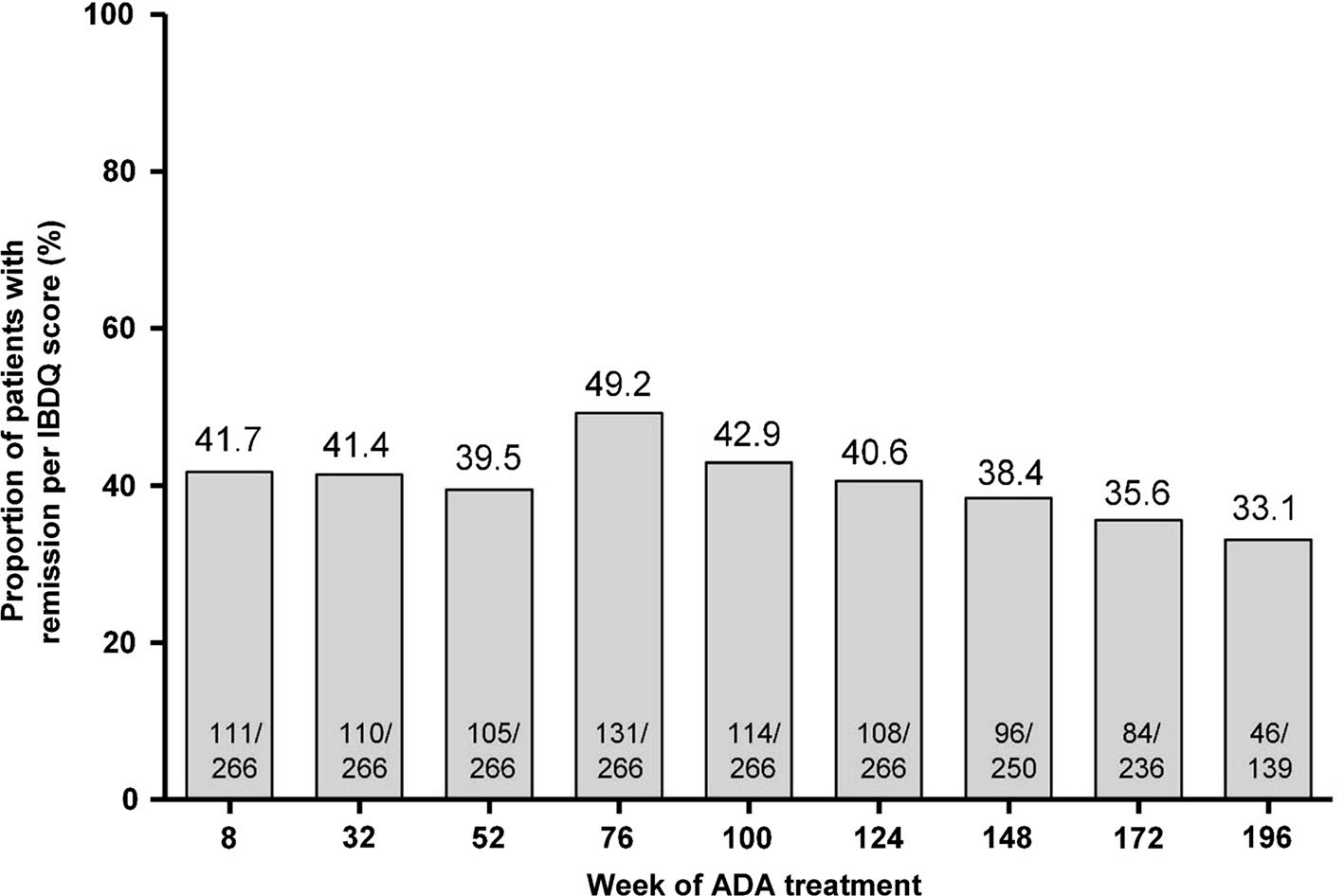
Proportion of patients in the “any ADA” set who achieved remission per the Inflammatory Bowel Disease Questionnaire (*IBDQ*) score (IBDQ score 170 points or greater) over time by duration of adalimumab (*ADA*) treatment (hybrid nonresponder imputation; *N* = 266)

Remission rates by the IBDQ score (IBDQ score 170 points or greater) remained stable from week 8 through week 196 of adalimumab treatment (Fig. 4). The mean change from the baseline in the SF-36 physical and mental component scores increased from week 8 through week 196 of adalimumab treatment (Fig. S2). Similarly, the mean change from the baseline in the IBDQ score increased over time from the first adalimumab dose to week 52 of treatment and was sustained up to week 196 of treatment (Fig. S3).

### Pharmacokinetics and immunogenicity

**Fig. 5:**
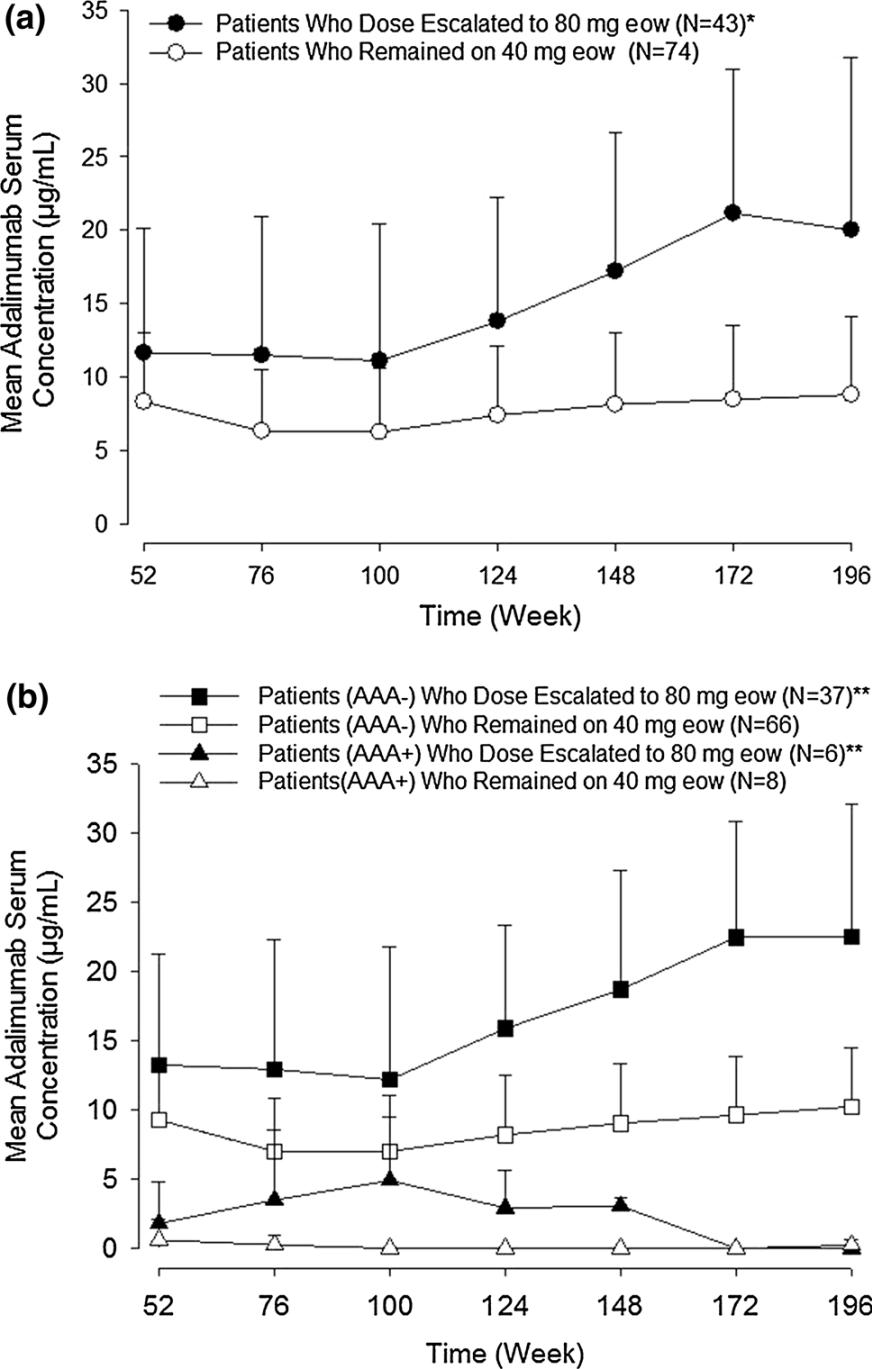
Adalimumab trough serum concentrations over time in patients who entered the open-label extension study **a** by dose-escalation status (continuing with 40 mg every other week vs escalation to 80 mg every other week) and **b** by dose-escalation status and anti-adalimumab antibody (*AAA*) status. The *error bars* denote standard deviation. *Single asterisk* median number of weeks of increased dose was 34 (range 15–155), *two asterisks* median number of weeks of increased dose was 31 (range 15–155) in AAA-negative patients and 79 (range 20–114) in AAA-positive patients, *eow* every other week

Serum adalimumab trough concentrations over time (from week 52 through week 196) for patients who were randomized to receive adalimumab in the lead-in study and entered the open-label extension study are presented by dose-escalation status (at any time) in Fig. 5a. Serum adalimumab concentrations in patients who continued to receive 40 mg every other week remained relatively constant from week 52 (mean ± standard deviation of 8.3 ± 4.7 µg/mL) through week 196 (8.8 ± 5.3 µg/mL). Concentrations increased in patients whose treatment was escalated to 80 mg every other week—from 11.7 ± 8.5 µg/mL at week 52 to 20.0 ± 11.7 µg/mL at week 196.

Sixteen of 190 patients (8.4%) were AAA positive during the open-label extension study, 10 of whom were AAA positive when they entered the study and 6 of whom became AAA positive after week 52. The mean serum concentration of adalimumab over time by AAA status and by dose-escalation status in the open-label extension study is shown in Fig. 5b. Serum adalimumab concentrations were higher in AAA-negative patients than in AAA-positive patients.

### Safety

Two hundred sixty-six Japanese patients with ulcerative colitis received one or more doses of adalimumab in the phase 2/3 study, representing 579.1 patient-years of exposure. An overview of treatment-emergent adverse events reported in the any ADA set is presented in Table 2.

**Table 2:** Treatment-emergent adverse events during adalimumab treatment for patients in the “any ADA” set (*N* = 266; 579.1 patient-years)

Adverse event	Events^b^
Any adverse event	2,499 (431.5)
Any adverse event at least possibly drug related	294 (50.8)
Severe adverse event	20 (3.5)
Serious adverse event	129 (22.3)
Adverse event leading to discontinuation of use of study drug	72 (12.4)
Infection	796 (137.5)
Serious infection	23 (4.0)
Opportunistic infection^a^	10 (1.7)
Tuberculosis	2 (0.3)
Ulcerative colitis worsening or flare	68 (11.7)
Injection site reaction	38 (6.6)
Hematologic adverse event	16 (2.8)
Allergic reaction	12 (2.1)
Hepatic adverse event	10 (1.7)
Any malignancy	8 (1.4)
Lymphoma	0
Nonmelanoma skin cancer	0
Intestinal stricture	5 (0.9)
Vasculitis	3 (0.5)
Pancreatitis	2 (0.3)
Psoriasis	2 (0.3)
Cerebrovascular accident	1 (0.2)
Interstitial lung disease	1 (0.2)
Erythema multiforme	1 (0.2)
Congestive heart failure	0
Demyelinating disorder	0
Lupus-like syndrome	0
Death	2 (0.3)

^a^Excluding oral candidiasis and tuberculosis

^b^The number of events per 100 patient-years is given in *parentheses*

The rates of any adverse events, possibly drug-related adverse events, severe adverse events, and serious adverse events were lower in the any ADA set than during the double-blind period. Also, the rates per 100 patient-years of infections, serious infections, opportunistic infections, tuberculosis, and adverse events leading to discontinuation of therapy were lower in the any ADA set than during the double-blind period.

The most frequent adverse event was worsening of ulcerative colitis. Malignancies were reported in eight patients, and all were judged to be unrelated to the study drug. No hepatic or hematologic adverse event required interruption or discontinuation of adalimumab treatment.

During treatment with adalimumab, tuberculosis was reported in two patients. Both events were considered by the investigator to be probably not related to the study drug. One of these patients, a 65-year-old man in the adalimumab 160/80 arm, died of tuberculosis on day 91 (62 days after the last dose). He had erythema larger than 10 mm but no induration on purified protein-derivative skin test at the baseline and was also receiving prednisone (20 mg/day) [18]. In addition, a 46-year-old man reported mild and nonserious *Mycobacterium tuberculosis* infection 50 days after the first adalimumab dose. Adalimumab therapy was discontinued on day 60 because of colon stenosis. Two deaths (one due to pancreatic carcinoma, the other due to tuberculosis, as described above) occurred during the double-blind period and were reported previously [18]. Both deaths were considered to be unrelated to the study drug. No deaths occurred during the open-label extension study.

In patients whose adalimumab dose was increased from 40 mg every other week to 80 mg every other week, the rates of any adverse events and of serious and opportunistic infections remained unchanged (Table S1). Most of the serious adverse events reported after dose escalation were related to ulcerative colitis.

## Discussion

This study has demonstrated the benefit of maintenance treatment with adalimumab for up to 4 years in Japanese patients with moderately to severely active ulcerative colitis. The remission rate per PMS was 30.8% (hNRI analysis) after 52 weeks of adalimumab treatment and was generally consistent throughout the open-label extension study up to week 196 (22.5%) of treatment. Similarly, the response rate per PMS (40.2% at week 52) was sustained through week 196 (32.5%) of adalimumab treatment.

The importance of mucosal healing as a therapeutic goal in ulcerative colitis (because of its association with favorable long-term outcomes such as long-term clinical remission, corticosteroid-free clinical remission, and avoidance of colectomy) has been demonstrated in several clinical studies, and was recently confirmed by a meta-analysis [19]. Clinically meaningful proportions of Japanese patients maintained mucosal healing up to 196 weeks of treatment (30.5%) in the present study. In addition, high proportions of patients who used corticosteroids at the lead-in study baseline were able to discontinue using steroids (69.0% at week 196 of treatment) and achieve steroid-free remission (40.5% at week 196 of treatment) with adalimumab treatment over time.

The long-term maintenance of efficacy of adalimumab therapy observed in Japanese patients is consistent with reports in Western patients. In the open-label extension study ULTRA 3, 24.7% of patients randomized to receive adalimumab at the study baseline achieved remission per PMS and 27.7% had mucosal healing after 4 years of therapy. Furthermore, approximately 60% of patients using corticosteroids at the lead-in study baseline were able to discontinue using corticosteroids at 4 years [17].

Approximately 40–50% of patients may not initially respond to anti-TNFα therapy, and about half of patients lose their response over time. However, the therapeutic value of dose escalation has been demonstrated in Western patients from ULTRA 2 who had lost their response to adalimumab, and these findings were confirmed in the present study in Japanese patients [20]. Allowing adalimumab dose escalation to 80 mg every other week in patients who experienced an inadequate response or disease flare resulted in higher rates of clinical response per PMS over time in the any ADA set than in patients who continued to receive 40 mg every other week.

Ulcerative colitis is associated with diminished quality of life, and a significant proportion of patients, particularly those with more severe symptoms, are unable to lead normal lives [21]. The current study has shown that the quality-of-life improvements observed in the original study were clinically meaningful and could be maintained for up to 4 years with long-term adalimumab administration.

No new safety signals were observed during the open-label extension study, and dose escalation to 80 mg every other week did not alter the safety profile of adalimumab. The safety of up to 4 years of treatment with adalimumab is consistent with the safety outcomes of the double-blind study [18] and other studies of adalimumab in ulcerative colitis [17] and other indications [22–24]. During the extension study, the exposure-adjusted incidence rates of adverse events, including serious adverse events, serious and opportunistic infections, malignancies, and hepatic and hematologic events, declined or remained similar to those in the 52-week double-blind period, thus providing evidence that there is no cumulative safety risk over time associated with long-term adalimumab therapy.

In AAA-negative patients who received adalimumab 40 mg every other week throughout their treatment, the mean serum adalimumab concentrations were constant for up to 4 years and were within the range of concentrations reported in Japanese Crohn’s disease patients who received the same dose. Consistently higher adalimumab concentrations were reported over time in patients with ulcerative colitis whose dose was escalated to 80 mg every other week than in dose escalators from the trial in Japanese patients with Crohn’s disease. In contrast, no noticeable difference in serum adalimumab concentration was identified in patients from both trials who continued to receive adalimumab 40 mg every other week [24]. An increase in adalimumab concentration after dose escalation is consistent with the higher response rates seen in the any ADA set than in the no-dose-escalation set reported above. Immunogenicity (one of more AAA-positive sample) developed in a small percentage of patients (8.4%) during the study, in line with data on development of immunogenicity in Japanese patients treated with adalimumab for Crohn’s disease (6.1%) [24].

The limitations of this study include its relatively small sample size, restriction to a Japanese population, and the open-label design of the extension period of the study. All patients who were recruited into the phase 2/3 study before the open-label extension study reported here were anti-TNFα naïve; hence this study cannot provide insights into the relative efficacy of adalimumab in anti-TNFα-naïve versus anti-TNFα-experienced patients. It would also be of interest to investigate how the results in this clinical trial setting compare with those in real-life clinical practice in Japan. A recent registry-based Spanish study reported a trend for higher 1-year response and remission rates with adalimumab therapy in anti-TNFα-naïve patients with ulcerative colitis compared with those previously treated with an anti-TNFα agent in routine clinical practice [25]. As with any long-term clinical study, there was loss of patient data over time due to early termination (e.g., because of lack of efficacy or adverse events). Another reason for discontinuation in our study is that adalimumab became commercially available during the study after its approval for the treatment of ulcerative colitis. Therefore, hNRI analysis was applied to binary efficacy end points, using stringent nonresponder imputation methodology until the latest possible time of observation of each enrolled patient and analysis of observed data thereafter. However, there are also several methodological strengths. The follow-up duration was extended to 4 years, the study was conducted according to strict quality standards compliant with good clinical practice, prospective data (including endoscopic and pharmacokinetic data) were systematically collected, and analyses of end points were prespecified.

In conclusion, this study has demonstrated that the beneficial effect of adalimumab for the treatment of moderately to severely active ulcerative colitis in Japanese patients is maintained through 4 years without the introduction of new safety signals.

## Electronic supplementary material

Below is the link to the electronic supplementary material.

Supplementary material 1 (PDF 471 kb)
