# Supplementary material for: Four-year maintenance treatment with adalimumab in Japanese patients with moderately to severely active ulcerative colitis
Source: J Gastroenterol. 2017 Mar 20;52(9):1031–40. doi: 10.1007/s00535-017-1325-2 (PMC5569655; doi:10.1007/s00535-017-1325-2)
Supplement: Supplementary file 1 — Supplementary material 1 (PDF 471 kb) [file 535_2017_1325_MOESM1_ESM.pdf]

Supplementary material

Fig. S1 Proportions of patients in the Any ADA set and No dose escalation set with (a) remission per PMS and (b) response per PMS, by duration of adalimumab treatment (hNRI; N = 266). *ADA* adalimumab, *PMS* partial Mayo score.

a

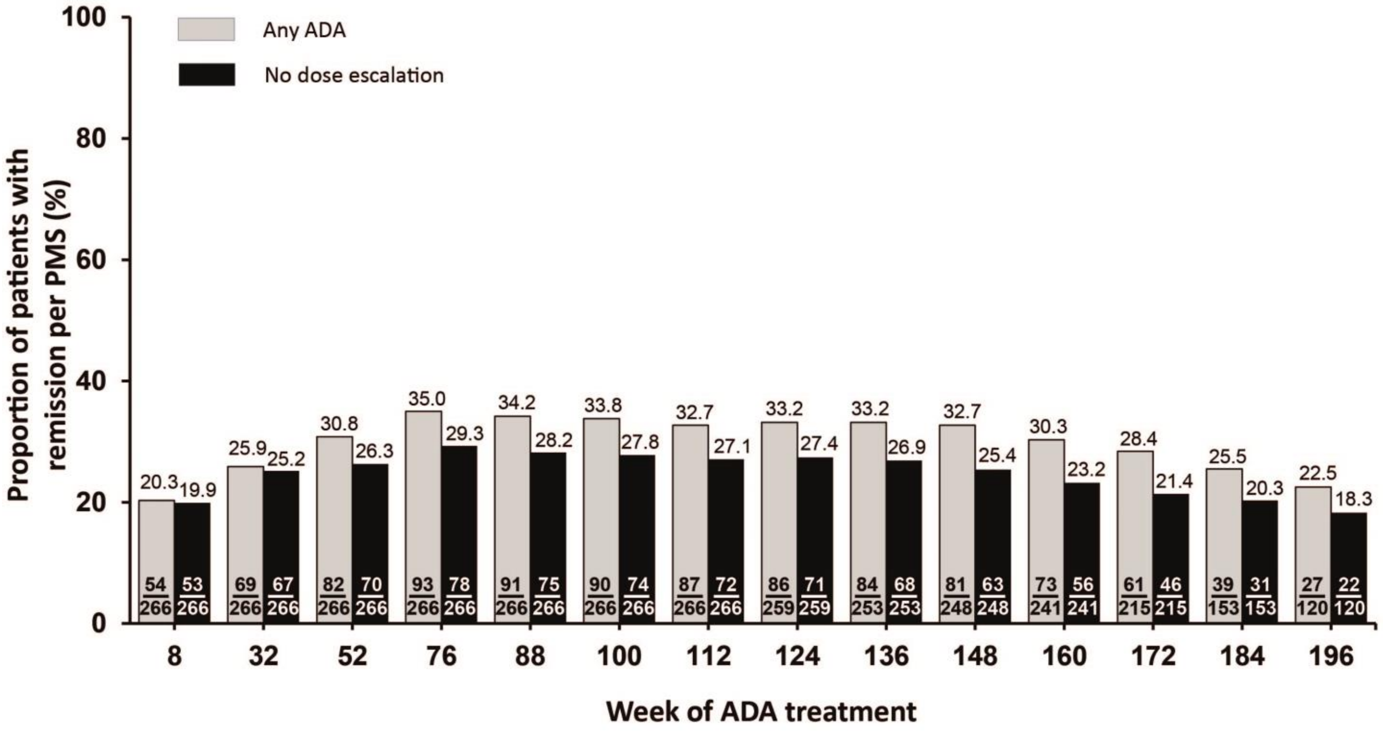

b

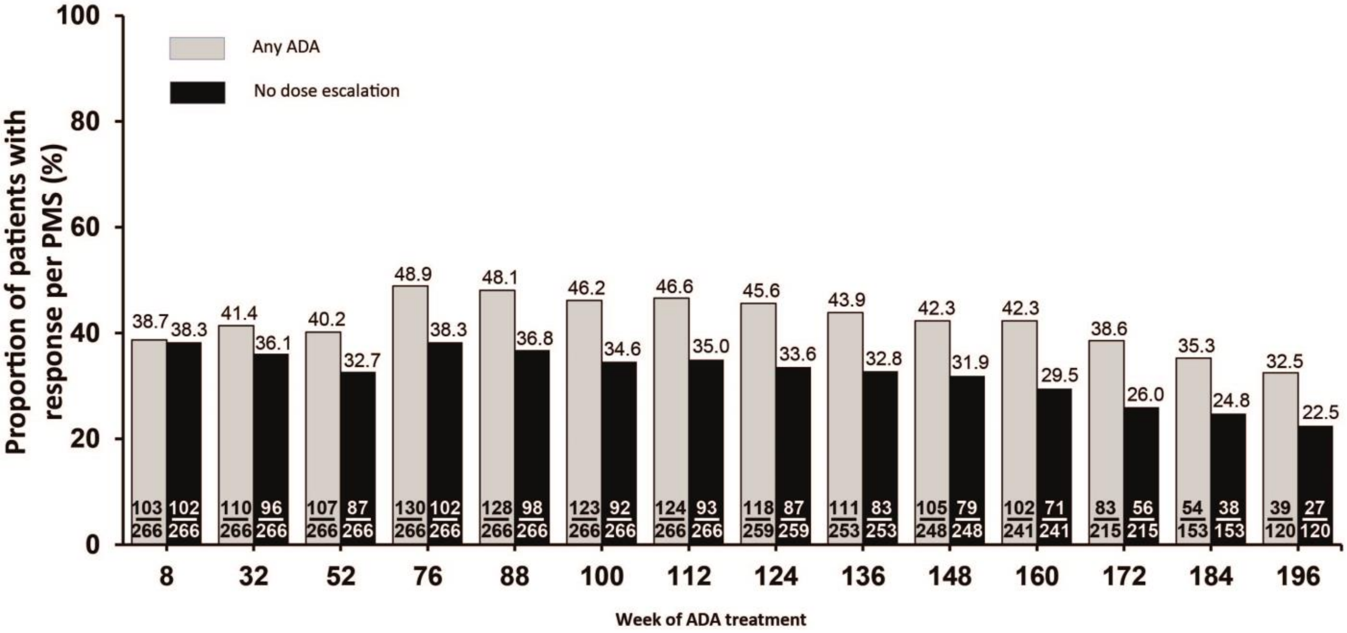
Any ADA
No dose escalation
Proportion of patients with response per PMS (%)
Week of ADA treatment
100
80
60
40
20
0
8
32
52
76
88
100
112
124
136
148
160
172
184
196
38.7
38.3
41.4
36.1
40.2
32.7
48.9
38.3
48.1
36.8
46.2
34.6
46.6
35.0
45.6
33.6
43.9
32.8
42.3
31.9
42.3
29.5
38.6
26.0
35.3
24.8
32.5
22.5
103/266
102/266
110/266
96/266
107/266
87/266
130/266
102/266
128/266
98/266
123/266
92/266
124/266
93/266
118/259
87/259
111/253
83/253
105/248
79/248
102/241
71/241
83/215
56/215
54/153
38/153
39/120
27/120

Fig. S2 Mean change from baseline in SF-36 summary scores in the physical and mental components by duration of adalimumab treatment for patients in the any ADA set (as observed). *ADA* adalimumab, *SF-36* 36-item short-form quality of life assessment.

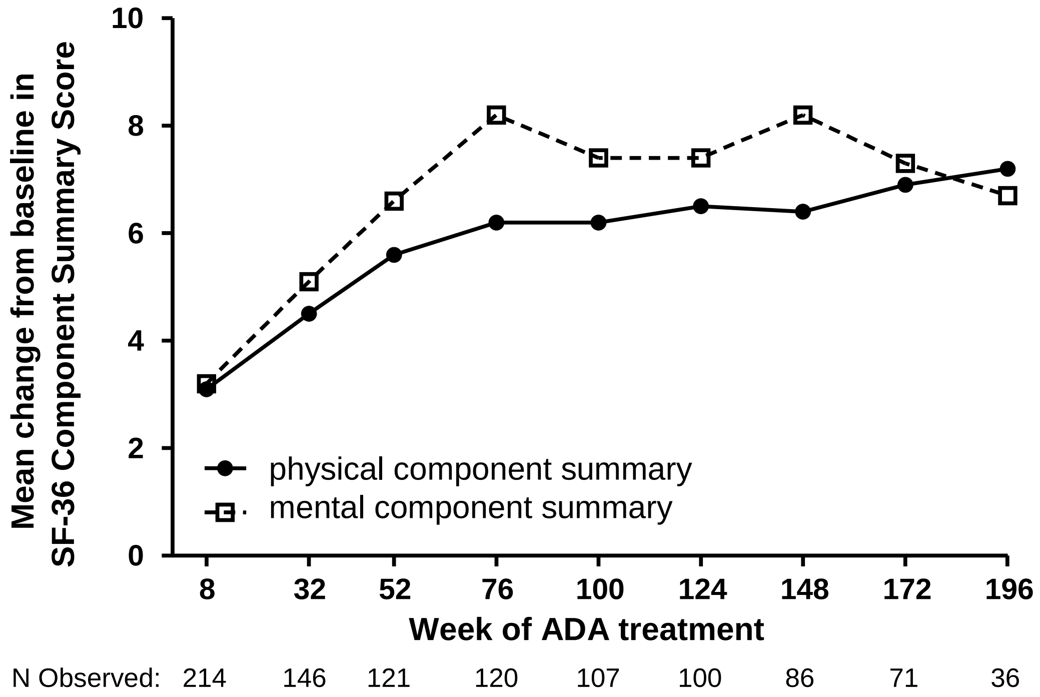

Fig. S3 Mean change from baseline in IBDQ score by duration of adalimumab treatment in patients in the any ADA set (as observed). *ADA* adalimumab, *IBDQ* Inflammatory Bowel Disease Questionnaire.

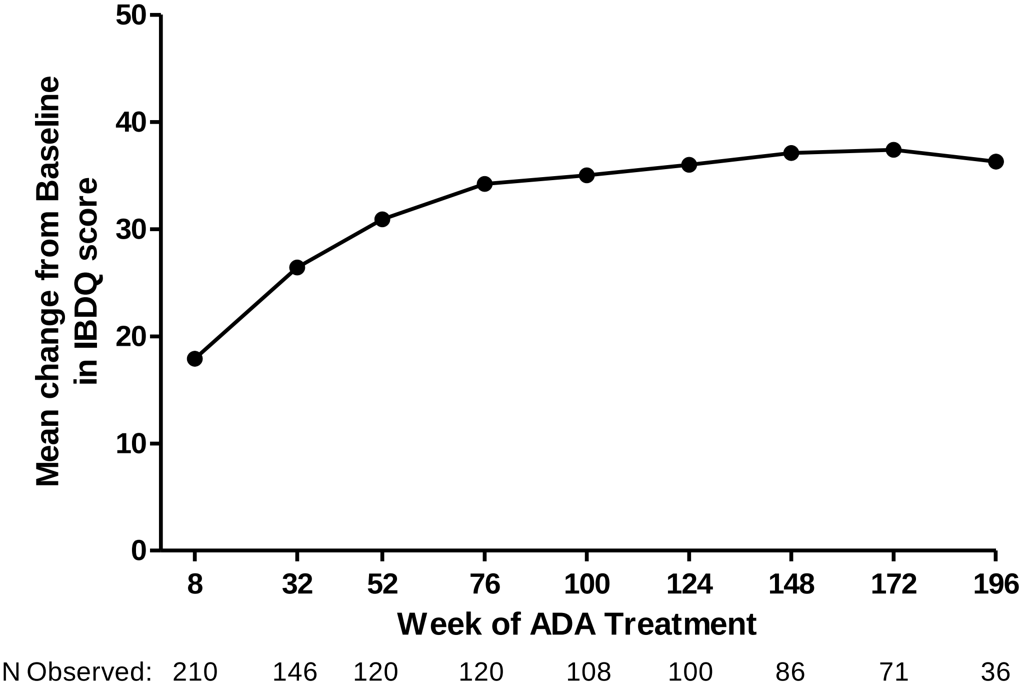

Table S1 Overview of AEs before and after dose escalation in patients in the any ADA set whose adalimumab dose was increased to 80 mg EOW

	Before dose escalation (N = 112, PY = 82.3) Events (E/100 PY)	After dose escalation (N = 112, PY = 139.6) Events (E/100 PY)
Any AE	349 (424.1)	611 (437.7)
Severe AE	2 (2.4)	5 (3.6)
Serious AE	12 (14.6)	49 (35.1)
AE leading to discontinuation of study drug	6 (7.3)	25 (17.9)
Infection	106 (128.8)	196 (140.4)
Serious infection	4 (4.9)	7 (5.0)
Opportunistic infection[a]	2 (2.4)	4 (2.9)
Tuberculosis	0	0
UC worsening or flare	13 (15.8)	28 (20.1)
Injection site reaction	9 (10.9)	3 (2.1)
Hematologic AE	3 (3.6)	3 (2.1)
Allergic reaction	2 (2.4)	0
Hepatic AE	4 (4.9)	1 (0.7)
Any malignancy	1 (1.2)	1 (0.7)
Lymphoma	0	0
Nonmelanoma skin cancer	0	0
Intestinal stricture	1 (1.2)	1 (0.7)
Vasculitis	1 (1.2)	1 (0.7)
Pancreatitis	0	0
Psoriasis	0	0
Cerebrovascular accident	0	1 (0.7)
Interstitial lung disease	0	1 (0.7)
Erythema multiforme	0	0
Congestive heart failure	0	0
Demyelinating disorder	0	0
Lupus-like syndrome	0	0
Death	0	0

ADA adalimumab, *AE* adverse event, *EOW*, every other week, *PY* patient-years, *UC* ulcerative colitis

[a] Excluding oral candidiasis and tuberculosis
